# The politics of health justice: Power, precarity and the fight for gender equality and SRHR

**DOI:** 10.1371/journal.pgph.0004599

**Published:** 2025-05-22

**Authors:** Rajat Khosla, Pascale Allotey

**Affiliations:** 1 Executive Director of the Partnership for Maternal, Newborn and Child, Health (PMNCH), World Health Organization, Geneva, Switzerland; 2 Director of the WHO Department of Sexual and Reproductive, Health and Research (SRH), which includes the UNDP/UNFPA/UNICEF/WHO/World Bank Special Programme of Research, Development and Research Training in Human Reproduction (HRP), World Health Organization, Geneva, Switzerland; PLOS: Public Library of Science, UNITED STATES OF AMERICA

Amartya Sen argued that health equity must grapple with broader questions of justice, fairness, and the social arrangements underpinning access to care [1]. The first months of 2025 have brought into question every aspect of this understanding of health equity. Cuts to global health funding, the breakdown of scientific cooperation, and the resurgence of regressive ideologies have not only undermined health systems but revealed the precarity of the global health architecture. Shifts in policy have already triggered the shutdown of HIV programmes across 50 countries, halted critical research, and suspended services for sexual and reproductive health. These are not isolated losses but structural signals of a broader retreat from justice. Sen reminds us that injustice lies not just in poor outcomes but in the denial of opportunity due to unequal social arrangements [2]. But what does this mean when those arrangements are shaped by actors thousands of miles away?

While the shrinking fiscal space rightly draws concern, this moment also demands deeper reflection. How did global health become so vulnerable to political shifts? And what would a justice-oriented global health system look like?

Among the most politically contested domains in global health is gender equality and sexual and reproductive health and rights (SRHR). The question is not merely why SRHR is under attack, but why it is so persistently targeted. The answer lies in the intersection of ideology, power, and patriarchy. These attacks are not aberrations; they reflect longstanding patterns seen in health crises from HIV to Ebola to COVID-19 [3]. Each time, rights, particularly for marginalized populations—are curtailed under pressure.

During the HIV/AIDS crisis, conservative agendas blocked access to life-saving services such as comprehensive sexuality education and harm reduction. Ebola responses often ignored the disproportionate impact on women and girls. COVID-19 exposed systemic failures again: increased gender-based violence, reduced access to contraception, and setbacks in reproductive rights [3].

The lesson is clear: sidelining SRHR weakens the robustness of health systems, exacerbates inequality, and reduces resilience. A justice-oriented system must anticipate these attacks and address the structural forces that allow them.

## Stop “accumulation” approach to global health

Koplan et al. defined global health as prioritizing health equity for all. Yet today, global health has drifted toward a model governed by “accumulation” approach —where decisions are based on projected returns, financial or otherwise. This commodification of health fails to confront the structural and historical barriers that perpetuate inequity [4].

Achieving gender equality in health means tackling the social, political, and economic barriers to access. However, market-driven models often reduce SRHR to transaction outcomes. With decline in funding in SRHR and de-prioritization of gender equality [5] there is a real risk of undermining fragile progress. For instance, in 2023, over 700 women died daily from preventable pregnancy-related causes. Majority of these deaths happen in countries which are often reliant on foreign aid [6]. Similarly, in 2021, 164 million women still lacked access to contraception vast majority of them live in LMICs [7].

Equity in health cannot be achieved through a market lens. Justice demands that we reject market logic in health policy and investment and reorient health policies and system to human rights.

## Re-thinking power

Power is a fundamental determinant of health, yet its role in policymaking is rarely interrogated. Decisions by powerful states and non-state actors disproportionately harm women, girls, and gender-diverse people. Anti-rights movements have gained ground, influencing negotiations and policy, and challenging the principles of justice, equity, and multilateralism [8].

Restrictions on SRHR are used to enforce control and uphold patriarchal norms. These measures harm the most marginalized people, regardless of geography. Institutional power imbalances and systemic discrimination limit states’ ability to meet legal and ethical obligations, resulting in rights rollbacks, rising maternal mortality, and unintended pregnancies.

Reimagining global health must include a rethinking of power: its sources, structures, and pathways. We need governance models that centre human capabilities and equity, not simply technical efficiency [9].

## Beyond aid, financing for gender equality and SRHR

Shrinking public health budgets, especially in aid-dependent countries, are deepening inequality. In some African nations, aid makes up 40% of health spending [10], and sudden cuts disrupt services and worsen outcomes in areas like maternal health, HIV, and TB. Sustainable, justice-based financing is crucial, with a shift toward domestic resource mobilization and resilient systems. While non-state actors play a role, concerns about accountability remain. Regional bodies like the African Union, BRICS, and G20 offer promising paths. AU initiatives such as the Maputo Protocol, CARMMA, and Africa CDC highlighting the power of regional leadership and South-South cooperation.

A shift from donor-dependence to equity-based, locally-driven financing is key to strengthen health services, including SRHR and sustaining health justice movements.

## Commitment to our values

A chilling effect is evident across global institutions, where fear of funding cuts has led to the erasure of references to diversity, equity, and inclusion. Some have capitulated to restrictive regulations, undermining access to safe abortion and other essential services.

But fundamental rights are not subject to political convenience. The Universal Declaration of Human Rights and the WHO Constitution both affirm health as a fundamental right. These principles have underpinned frameworks from Alma-Ata to today. In moments of regression, they must be upheld, not abandoned.

## Re-aligning our movements

The fight for gender equality and SRHR cannot be separated from broader struggles for justice—whether in climate, trade, or economic policy. Now more than ever, we must embrace intersectionality and build alliances across movements to confront systemic inequalities fueling these crises.

However, global health remains dominated by elite structures that exclude diverse voices, particularly from the Global South. Real progress requires investing in grassroots movements and ensuring that those most affected by health inequities have a seat at the table. Strengthening coalitions, fostering cross-regional partnerships, and dismantling entrenched power imbalances are essential steps toward a more just and inclusive global health agenda.

To tackle growing SRHR and global health challenges, we need to move away from market-driven models that commodify health and instead adopt equity- and rights-based approaches. This means dismantling political and patriarchal power structures that marginalize women, girls, and gender-diverse people. Sustainable, locally driven financing is key, as is resisting anti-rights movements and protecting gender equality. Progress depends on intersectional, grassroots coalitions that center affected communities in shaping policy and justice efforts.

To move forward in these challenging times requires bold action, unwavering solidarity and a steadfast commitment to justice.
